# ﻿Types matter: taxonomic status of *Chanodichthys oxycephalus* (Bleeker, 1871) (Cypriniformes, Xenocyprididae) and some relevant species of East Asia

**DOI:** 10.3897/zookeys.1257.120512

**Published:** 2025-10-30

**Authors:** Xiao Chen, Shiping Su, Xiaohua Zhang, Richard Van der Laan, E Zhang

**Affiliations:** 1 College of Animal Science & Technology, Anhui Agricultural University, Hefei, China Anhui Agricultural University Hefei China; 2 Institute of Hydrobiology, Chinese Academy of Sciences, Wuhan, China Institute of Hydrobiology, Chinese Academy of Sciences Wuhan China; 3 Almere, Netherlands Unaffiliated Almere Netherlands

**Keywords:** Cultrin fishes, nomenclature, species recognition, taxonomic chaos

## Abstract

Issues concerning the identification of *Chanodichthys oxycephalus* are addressed. This species is a senior synonym of *Ch. oxycephaloides* and is restricted to the Chang-Jiang basin and adjacent lakes. Specimens previously identified as *Ch. oxycephalus*, represent misidentifications of either *Ch. abramoides* or *Ch. dabryi*. *Chanodichthys oxycephalus*, *Ch. abramoides*, and *Ch. dabryi* are confirmed as three distinct species. *Chanodichthys dabryi shinkainensis* is treated as a junior synonym of *Ch. abramoides*. Additional comments are provided on some cultrin fishes from Lakes Dongting and Xingkai mentioned in earlier literature. This study clears up the taxonomic confusion surrounding associated species.

## ﻿Introduction

Cultrin fishes represent a group of xenocypridids characterized by a remarkably compressed body with distinct ventral keel, elongated anal fin, and semi-buoyant or adhesive egg-laying behavior in lentic habitats and widely distributed across the river-lake systems of East Asia (Chen et al. 2022; Cheng et al. 2022). This group includes many genera of economic significance in China, such as *Chanodichthys* Bleeker, 1860, *Culter* Basilewsky, 1855, *Megalobrama* Dybowski, 1872, and *Parabramis* Bleeker, 1864 (Tang et al. 2013). It has been shown that the vast majority of East Asian cultrin fishes have a relatively short evolutionary history (18.5–0.2 Mya) (Chen et al. 2022a; Cheng et al. 2022; Van der Laan 2025). Although phylogenetic relationships of these fishes are recently evaluated with molecular evidence (Wang et al. 2007; Tang et al. 2013; Chen et al. 2022a), their generic classification and species identification are not fully clear.

Currently, the genus *Chanodichthys* comprises ten species: *Ch. abramoides*, *Ch. dabryi*, *Ch. elongatus*, *Ch. erythropterus*, *Ch. flavipinnis*, *Ch. mongolicus*, *Ch. oxycephaloides*, *Ch. oxycephalus*, *Ch. qionghaiensis*, and *Ch. recurviceps* (Chen et al. 1998; Fricke et al. 2025), while *Culter* consists of two species, *Cu. alburnus* and *Cu. compressocorpus* (Kottelat 2013; Fricke et al. 2025). All these species are widespread in Russia, Korean Peninsula, and China, with an extended distribution of a single species *Ch. flavipinnis* to northern Vietnam (Appendix 1: Table A1). Most of them, being of economic importance, are commonly encountered in catches of rivers and lakes fisheries (Peng et al. 2009; Wang et al. 2017). The two genera have been the subjects of modern taxonomic research on cultrin fishes (Yih and Chu 1959; Bănărescu 1967a, 1967b, 1972; Li 1992; Luo 1994; Luo and Yue 1996). Previous molecular phylogenetic studies indicated that the generic classification and species identification of the subfamily Cultrinae, as conventionally defined (Luo and Chen 1998), might be inaccurate (Wang 2007; Tang et al. 2013; Chen et al. 2022a).

*Chanodichthys oxycephalus* seems to be an elusive fish. It was initially described in *Culter* by Bleeker (1871) based on a single specimen of 290 mm SL collected from the Yang-tse-kiang or Yangtze River (today’s Chang-Jiang in mandarin Chinese) by Dabry de Thiersant, a French consular official in China (Luo 2005). Taking into account that the consulate where Dabry then served was located in Hankau (today’s Hankou), Hubei Province, central China (Israeli 1989), its type locality might be the Hankou District of Wuhan City, in the middle Chang-Jiang basin. Subsequent researchers referred this fish to *Erythroculter* and reported on its occurrence in Lake Shinkai, the Ussuri River of the lower Amur River basin, and the Chang-Jiang basin (Yih and Chu 1959; Wu 1964). It was later transferred to *Culter* (Luo and Chen in Chen et al. 1998; Zhang and Zhao 2016), and recently to *Chanodichthys* (Kottelat 2013; Chen et al. 2022c). Despite its current recognition as a valid species in Chinese literature, the current systematics of *Ch. oxycephalus* still remain poorly understood. No additional specimens have been caught from its known range since Yih and Chu (1959). During two decades of our fish surveys in the mid-lower Chang-Jiang basin, lots of collected specimens without exception were identified as *Ch. oxycephaloides* rather than *Ch. oxycephalus*. When Chinese *Culter* (now transferred to *Chanodichthys*) species were taxonomically revised, no researcher examined the type specimen of *Ch. oxycephalus*. To address the issue regarding the validity of *Ch. oxycephalus*, photographic examination of the types was performed for this species and some relevant species. Also, over one hundred specimens, provisionally identified as *Ch. oxycephaloides* or *Ch. oxycephalus* from the Chang-Jiang basin, were examined. Seventy-one curated specimens from other basins (including Lake Xingkai, Heilongjiang Province) were examined to clarify the ambiguity between *Ch. oxycephalus* and *Ch. Oxycephaloides*, and the relationship with some other relevant cultrin fishes. A taxonomic explanation for the lack of official sampling records of *Ch. oxycephalus* for more than 60 years is provided here.

## ﻿Material and methods

### ﻿Specimen sampling and preservation

Specimens utilized for this study were sampled in accordance with the Chinese Laboratory Animal Welfare and Ethics animal welfare laws (GB/T 35892-2018). Gill nets and trap nets were used to collect specimens. After being anesthetized, all captured individuals were fixed by immersion in either ethanol or formalin. For morphological examination, caught specimens of cultrin fishes were stored in 10% formalin. Voucher specimens are deposited in the collection of the Museum of Aquatic Organisms at the Institute of Hydrobiology, Chinese Academy of Sciences (IHB).

### ﻿Morphological analysis

Measurements were taken point to point with digital calipers connected directly to a data-recording computer via Bluetooth and data recorded to the nearest 0.1 mm. Measurements were taken on the left side of specimens whenever possible, following methods used by Kottelat (2001) and Xie et al. (2003). Morphological examinations were made in this study for type specimens deposited in the relevant museums (see abbreviations in Appendix 1: Table A2). Head length (HL) and measurements of other parts of the body are given as percentages of standard length (SL) (Table 1). Counts of vertebrae were taken from radiographs of Micro-CT or X-rays.

**Table 1.** Morphometric measurements and meristic counts for *Chanodichthys oxycephalus*, *Ch. abramoides*, and *Ch. dabryi*. Note: SD (standard deviation).

Character	*Ch. oxycephalus* (n = 30)		*Ch. abramoides* (n = 21)		*Ch. dabryi* (n = 23)	
	Range	Mean ± SD	Range	Mean ± SD	Range	Mean ± SD
SL (mm)	66.7–236.7	136.9 ± 52.4	149.8–270.9	208.6 ± 30.9	73.7–193.5	140.5 ± 35.0
**Morphometric data**						
% **of SL**						
Body depth	22.2–29.5	25.5 ± 1.9	25.9–31.3	27.9 ± 1.7	21.0–27.9	24.5 ± 1.7
Body width	8.0–11.9	9.4 ± 1.1	7.7–9.5	8.9 ± 0.5	8.5–11.8	9.5 ± 0.9
Caudal-peduncle length	9.2–15.4	12.0 ± 1.8	10.9–14.4	12.2 ± 1.1	8.0–13.9	11.6 ± 1.5
Caudal-peduncle depth	6.7–10.9	8.9 ± 1.1	8.4–10.8	9.7 ± 0.6	8.2–11.2	9.3 ± 0.8
Pectoral-fin length	15.0–20.2	16.7 ± 1.6	18.2–21.8	19.7 ± 1.0	17.9–20.8	19.2 ± 0.9
Pelvic-fin length	12.9–17.9	14.7 ± 1.3	15.5–19.7	17.4 ± 1.1	15.8–19.3	17.5 ± 0.9
Anal-fin length	11.0–13.3	12.3 ± 0.8	21.6–27.0	24.9 ± 1.6	9.6–13.9	12.4 ± 1.0
Dorsal-fin length	16.8–23.5	21.1 ± 2.3	18.3–23.0	20.5 ± 1.6	14.2–24.2	19.7 ± 2.4
Head length	25.2–29.2	26.7 ± 1.2	25.2–29.6	27.0 ± 1.2	23.9–26.9	25.6 ± 0.9
% **of HL**						
Head depth	45.6–57.8	51.5 ± 3.5	67.4–84.2	79.8 ± 4.6	52.7–63.9	58.1 ± 2.6
Head width	32.5–40.6	36.2 ± 2.5	33.7–42.3	37.5 ± 2.4	34.6–45.0	39.0 ± 2.3
Snout length	26.1–31.0	28.5 ± 1.6	26.4–32.6	29.6 ± 2.1	23.2–32.3	27.9 ± 2.0
Eye diameter	17.6–28.9	21.1 ± 3.3	16.1–36.1	19.4 ± 5.0	18.3–27.2	21.8 ± 2.6
Mouth depth	21.8–39.5	30.3 ± 7.2	30.4–55.0	35.0 ± 6.2	25.7–39.9	31.5 ± 3.8
Mouth width	60.4–67.8	65.0 ± 2.4	63.7–87.8	73.5 ± 7.2	51.9–75.1	60.7 ± 4.9
**Meristic counts**						
Lateral-line scales	73–75	74 ± 0.5	64–69	68 ± 0.5	64–70	67 ± 0.7
Scale rows above lateral line	13	13	12–13	12 ± 0.5	13–14	13 ± 0.5
Scale rows below lateral line	7	7	8–9	8 ± 0.5	6–7	6 ± 0.5
Circum-peduncular scales	21–22	21 ± 0.5	20–23	22 ± 0.5	20–22	21 ± 0.5
Vertebral counts	4+37–38	37 ± 0.5	4+42–43	42 ± 0.8	4+41–43	42 ± 0.3

## ﻿Results

### ﻿Redescription of *Chanodichthys oxycephalus* (Bleeker, 1871)

#### ﻿ Chanodichthys oxycephalus

Taxon classificationAnimaliaCypriniformesXenocyprididae

(Bleeker, 1871)

AD0CE641-D8E2-5F78-AFB9-8E9F1330C2B5

Culter oxycephalus Bleeker, 1871a: 74, pl. 5 (Chang-Jiang, China). Appeared first as name only in Bleeker 1870: 252. Also appeared in Bleeker 1871b: 87 and Bleeker 1873: 10.
Culter oxycephaloides Kreyenberg & Pappenheim, 1908: 104 (Lake Dongting, south central China); Compilation Group XNC 1984: 53–54 (Xichuan); Yang 1987: 61–62 (Lake Liangzi); Wu 1989: 66–67 (Chishui); Institute SFR and Department of Biology SNU 1992: 44–45 (Xunyang, Xixiang, Baihe, Ankang); Luo 1994: 47 (Chang-Jiang); Chen et al. 1998 (mid-upper Chang-Jiang basin); Yao 2010: 37 (Anqing, Chaohu, Chang-Jiang); Guo et al. 2021: 164–167 (Guangyuan, Nanchong, Yibin); Wu et al. 2021: 98–99 (Xinhua, Hengyang); Wang 2022: 128 (Nanjing); Chen and Fu 2024: 122–123 (Xinjian, Duchang, Poyang).
Erythroculter oxycephaloides : Nichols 1928: 30 (Lake Dongting); Yih and Chu 1959: 87 (Lake Liangzi); Wu 1964: 103 (Lake Liangzi, Yunxian, Mudong, Hechuan).
Culter (Erythroculter) oxycephaloides : Kimura 1934: 107 (Chongqing).

##### Specimens examined.

• MNHN 0000-5050, ***holotype***, 290 mm SL: Chang-Jiang; • ZMB 16686, 172 mm SL: Lake Dongting (photographic examination); • IHB 201807055611–14, 8 specimens, 65.9–122.4 mm SL: Hunan Province: Xiangyin County, Menggu Village (28°48'04.88"N, 112°53'28.42"E), caught by X. Chen, C. An, W. Shao, 5 Jul 2018; • IHB 201805055611–14, 3 specimens, 65.9–122.4 mm SL: Hunan Province: Yueyang City, Chenglingji (29°26'09.73"N, 113°08'43.87"E), collected by X. Chen, D. T. Nguyen, L. Zhang, 10 May 2018. • IHB 201807165611–14, 4 specimens, 65.9–122.4 mm SL: Hunan Province: Yueyang City, Hongqihu (29°13'56.86"N, 112°57'11.99"E), collected by X. Chen, L. Cao, L. Qiu, 16 Jun 2018.

##### Diagnosis.

*Chanodichthys oxycephalus* can be diagnostically separated from all congeneric species by its reduced vertebral count (4+37-38), and distinguished from other species, except morphologically similar congeners *Ch. dabryi* and *Ch. abramoides*, by having a sub-superior and oblique mouth and 23–29 branched anal-fin rays. It is distinct from the latter two species in having a pointed (vs non-pointed), steeply (vs –gradually) elevated humpback behind to the head and higher lateral-line pored scales 73–75 (vs ≤ 70).

##### Description.

**Figure 1.**
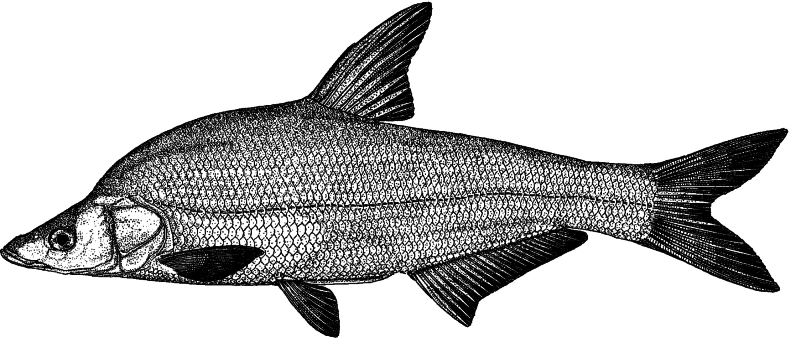
Lateral view of *Chanodichthys oxycephalus*, ca 250 mm TL; China: Jiangsu: Nanjing, Jul 2021, collected by Tao Ju and Zhaochen Ding. Illustrated by Zhixian Sun.

Morphometric measurements for examined specimens in this study provided in Table 1. See Fig. 1 for general appearance.

Body strongly compressed and deep; dorsal profile convex with a significant hump posterior to nape and ventral profile somewhat straight. Abdominal keel developed from the pelvic-fin insertion to the anus. Head small, pointed, laterally compressed, length less than body height. Snout protruded, longer than eye diameter. Mouth sub-superior, slit; maxilla slightly shorter than mandible, with its posterior end extending backwards below nostril but not to anterior margin of orbital. No barbels. Eye large, laterally positioned in anterior half of head. Interorbital space wide and slightly convex, distance larger than eye diameter. Nostril near anterior margin of eye, with lower margin above a line aligning upper margin of eye. Gill aperture broad, extending forward approximately below posterior margin of eye. Gill membranes united to narrow isthmus.

Dorsal fin with 3 simple and 7–8 branched rays; last simple ray stiff with a smooth posterior margin, and shorter than HL; origin posterior to vertical through pelvic-fin base; distal margin slightly concave. Pectoral fins short and pointed, with 1 simple and 15–16 branched rays; tip of adpressed fin rays not reaching pelvic-fin insertion. Pelvic fin with 2 simple and 8 branched rays, inserted anterior to dorsal-fin origin, or midway between pectoral-fin insertion and anal-fin origin; tip of adpressed fin rays not reaching anal-fin origin. Pelvic axillary scale present, short, not reaching beyond base of last ray. Anal fin with 3 simple and 23–26 branched rays; origin posterior to vertical through posterior end of dorsal-fin base, or much closer to pelvic-fin base than to caudal-fin base, distal margin slightly concave. Caudal fin deeply forked, longest rays more than twice as long as shortest rays, and upper and lower lobes pointed.

Lateral line complete, originating from upper extremity of gill opening, descending downwards above pectoral-fin base, and extending almost straightly along the mid-lateral of body, running parallel to the ventral margin onto caudal peduncle. Perforated scales 73 (14) or 75 (16); scale rows above lateral line 13 (30) and below 7 (30); circum-peduncular scales 21 (16) or 22 (14) and pre-dorsal scales 13 (30).

##### Coloration.

In freshly collected specimens, head and dorsum of body grey-black, underside and abdomen silver; back and lateral head peppered with small dark spots. Back darker and belly lighter. Fins reddish, caudal fin orange-red.

In formalin-stored specimens, ground color slightly faded; body dorsally greyish and ventrally greyish-white and back of head becoming yellowish-brown. Fins grey to creamy yellow.

##### Sexual dimorphism.

No sexual dimorphism was observed in the specimens checked.

##### Geographical distribution and habitat.

**Figure 2.**
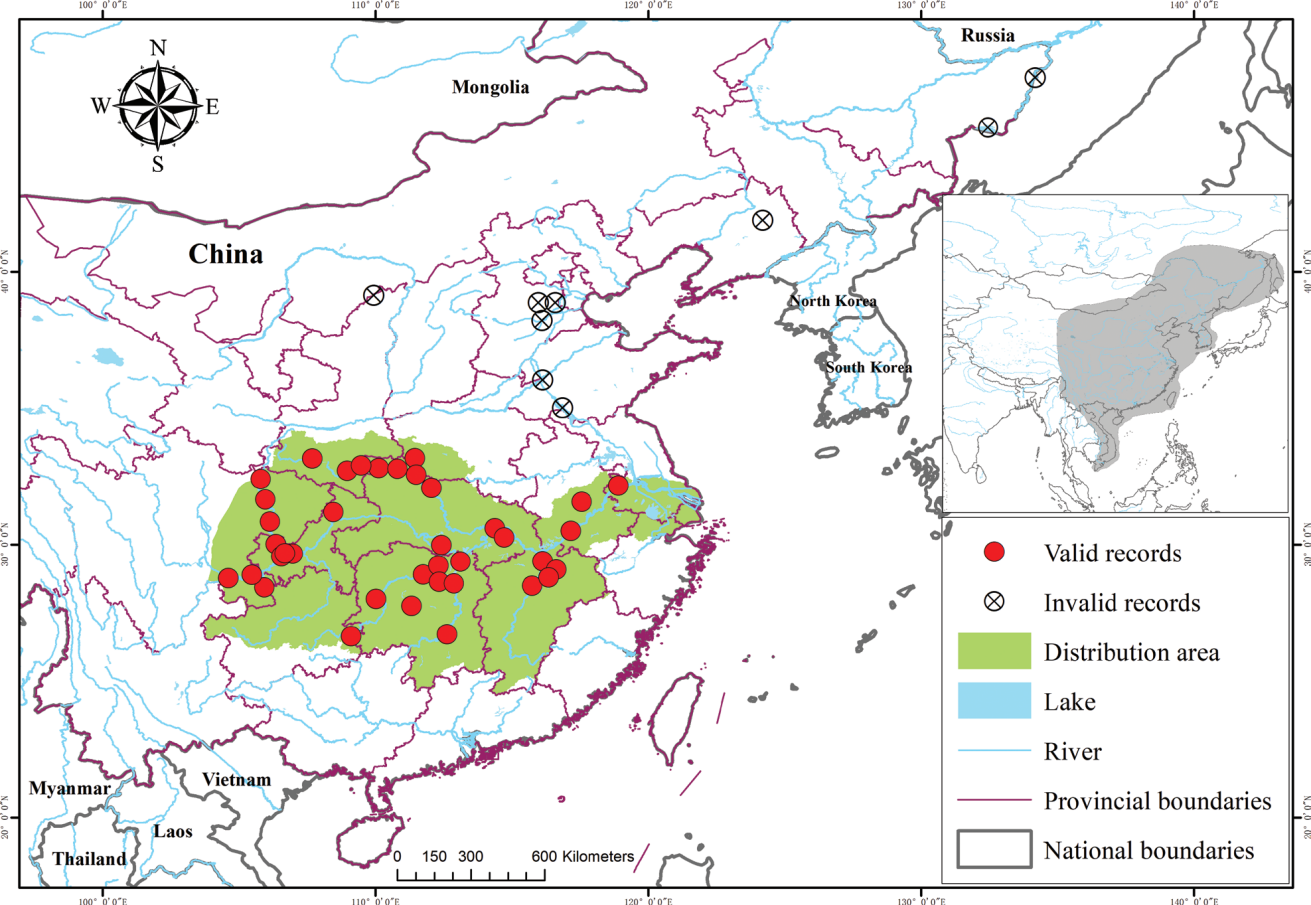
Distribution map of *Chanodichthys oxycephalus* (green area) and related records compiled from historical literature and field surveys. Note: red solid circles = valid records; crossed circles = literature-derived invalid historical records. The grey region delineates the sympatric distribution of cultrin fishes, viz., the studied area.

*Chanodichthys oxycephalus* is restricted to the Chang-Jiang basin (Fig. 2), based on the data obtained during the field survey and historical records from the literature (Compilation Group XNC 1984; Yang 1987; Wu 1989; Institute SFR and Department of Biology SNU 1992; Yao 2010; Duan et al. 2015; Guo et al. 2021; Wu et al. 2021; Wang 2022; Chen and Fu 2024). Specimens, previously identified by Luo and Chen (1998) as *Culter oxycephaloides* from the mid-upper Chang-Jiang basin, belong to this species; meanwhile, the voucher specimens, recognized by them as *Ch. oxycephalus* from Lake Liangzi of Hubei Province and Lake Shinkai of Heilongjiang Province, are in fact a misidentification of *Ch. dabryi*.

## ﻿Discussion

### ﻿Identity of *Chanodichthys oxycephalus*

**Figure 3.**
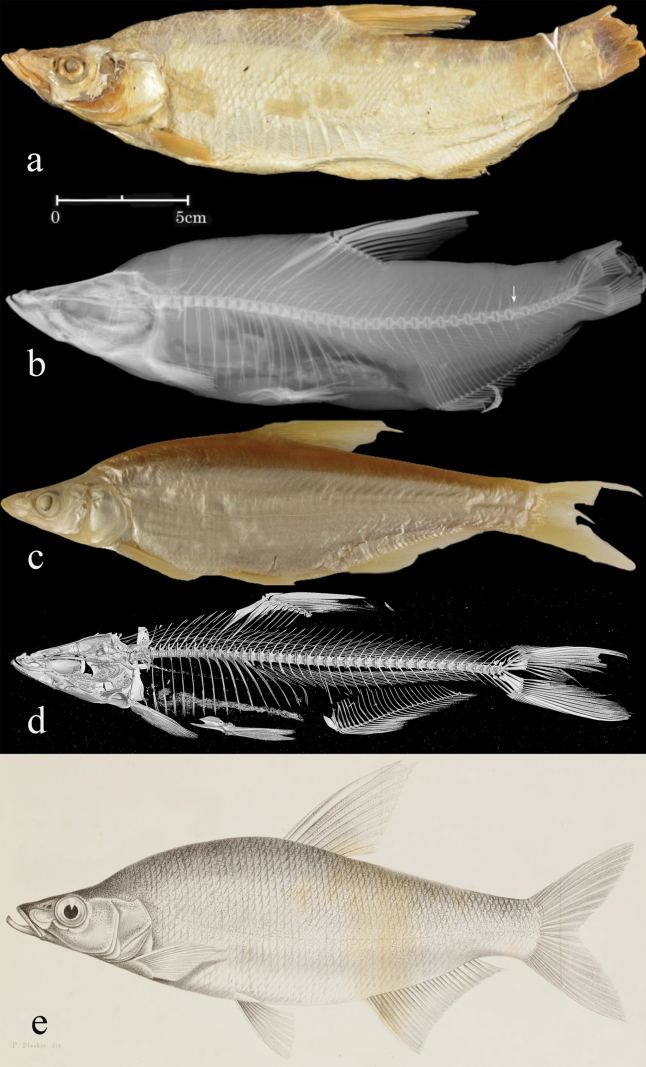
a, b. *Chanodichthys oxycephalus*, MNHN 0000-5050, holotype; a. Lateral view. b. X-ray image, arrow indicates the abnormal vertebrae; c, d. *Ch. oxycephaloides*, ZMB 16686, holotype; c. Lateral view; d. Micro-CT photograph; e. Bleeker’s (1871a) illustration of *Ch. oxycephalus*.

The *Ch. oxycephalus* reported in Chinese literature has long been misidentified. Specimens, so far identified as *Ch. oxycephalus* from Lake Liangzi of Hubei Province and Lake Xiaoxingkai of Heilongjiang Province (Luo and Chen 1998: 193, fig. 112), do not conform to the species depicted by Bleeker’s (1871a) illustration of its holotype in terms of head shape and dorsal spine length. Our photographic examination on the holotype of *Ch. oxycephalus* (MNHN 0000-5050, Fig. 3a, b), which is not in a good state, indicates that it has at least 73 lateral-line pored scales, and 22 scale pockets likely corresponding to the same count of circum-peduncular scales, both more numerous than the 65, as stated in Bleeker’s (1871a) description. Thus, these numbers indicate that the original description was inaccurate in these two meristic counts. This inaccuracy is also seen in his accompanying drawings of several other fish species, for example, the lateral line was missing in pl. 2 fig. 2 for *Acanthorhodeus macropterus* (= *Acheilognathus macropterus*), incorrectly counted in pl. 6 fig. 1 for *Hemibarbus dissimilis* (= *Paracanthobrama guichenoti*), and indistinctly depicted in pl. 4: fig. 2 for *Sarcocheilichthys sinensis*. Main reasons for this might be: (1) the lateral-line scales were then believed to be of no taxonomic importance for species identification, thus receiving little or no attention, and (2) the lower counts of lateral line scales likely resulted from the ease with which cultrin fish scales are shed.

**Figure 4.**
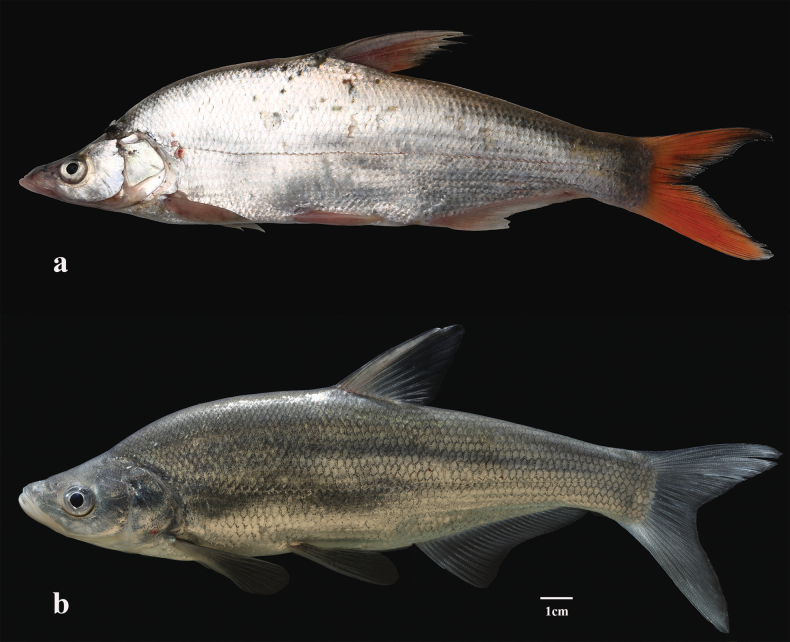
a. Lateral view of *Chanodichthys oxycephalus*, China: Hunan, Xiangyin County, Lake Dongting, collected by X. Chen, L. Cao, L.H. Qiu, photographed by C.T. An, 26 Oct. 2017; b. *Ch. dabryi*, China: Hunan, Miluo County, Lake Dongting, collected by X. Chen, L. Cao, Z.T. Wang, photographed by C.T. An, 20 Jan. 2018.

Actually, *Ch. oxycephalus*, as here defined, is a subjective senior synonym of *Ch. oxycephaloides*. Kreyenberg and Pappenheim’s (1908) description of *Ch. oxycephaloides* as a new species was mainly based on its differences in the count of lateral-line scales with *Ch. oxycephalus*, without examination on its holotype. The *Ch. oxycephaloides* of Chinese literature (e.g. Luo and Chen 1998: 197, fig. 115) conforms to its original description. This fish, as depicted in Bleeker’s (1871a) illustration of the holotype, has a pointed snout in lateral view, a small head with large eyes and a straight dorsal profile, a prominent humpback behind the head, and pectoral fins inserted vertically anterior to the dorsal-fin origin, and 65 lateral line pored scales. Except the last count, all these characters are completely in agreement with those of *Ch. oxycephaloides* based on our photographic examination on the holotype (ZMB 16686, Fig. 3c, d). As noted above, lateral line pored scales are at least 73 for the holotype of *Ch. oxycephalus*, marginally aligning with the known range of this count (73–85) for *Ch. oxycephaloides* (Luo and Chen 1998: 196). The same species is thus represented by the holotypes of *Ch. oxycephalus* and *Ch. oxycephaloides*, and their type localities are in the middle Chang-Jiang basin (Fig. 4a).

**Figure 5.**
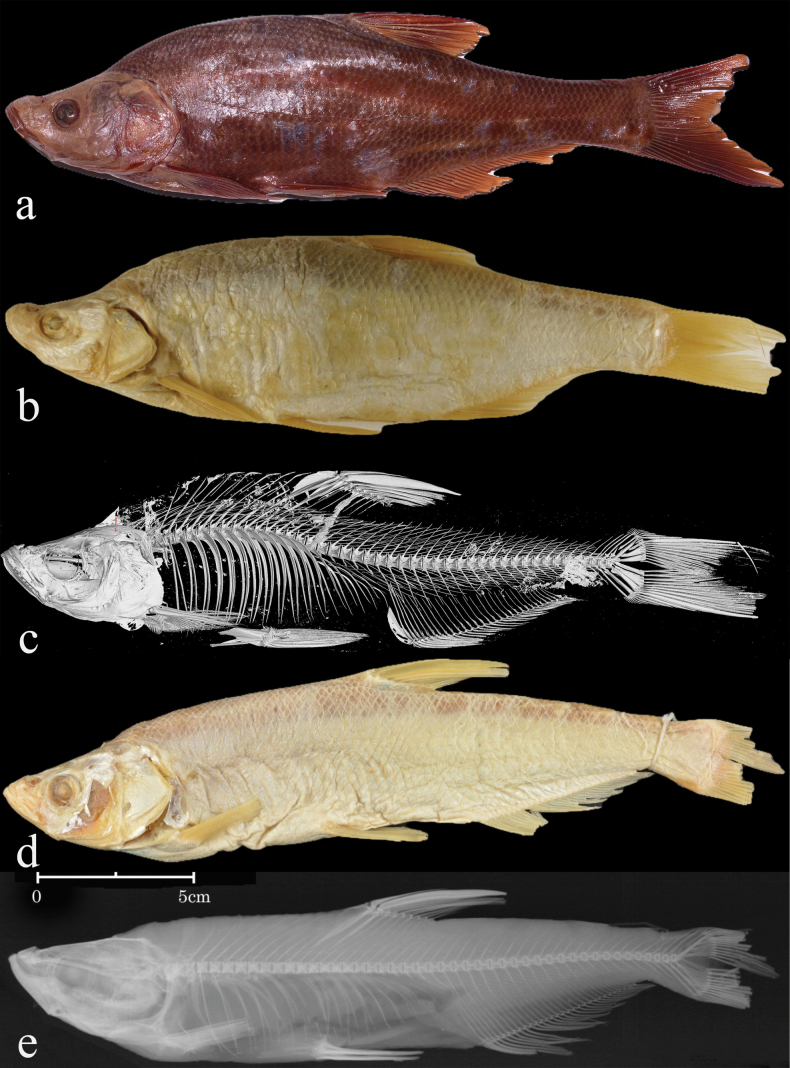
a. Lateral view of *Erythroculter dabryi shinkainensis*, IHB 58-1558, syntype; b. *Chanodichthys abramoides*, ZMB 7933, syntype; c. Micro-CT image of *Ch. abramoides*; d. *Ch. dabryi* MNHN 0000-5078, holotype; e. X-ray image of *Ch. dabryi*.

It is essential to clarify the taxonomic confusions surrounding the misidentification of *Ch. oxycephalus*. This species, as here defined, is so far known from the Chang-Jiang basin and affiliated lakes. Our examination indicated that three specimens, under the name of *Ch. oxycephalus* from Lake Liangzi of Hubei Province and Lake Shinkai of Heilongjiang Province, China (Luo and Chen in Chen et al. 1998), have a sub-superior mouth, fewer than 70 lateral-line pored scales, and a blunt snout and a straight dorsal profile of the head. These characters render them conspecific with *Ch. dabryi* of Chinese authors. *Chanodichthys dabryi* was originally described by Bleeker (1871a) based on a single specimen of 270 mm SL, caught in 1868 by Dabry de Thiersant from the Chang-Jiang. It is currently identified as a valid widespread species in China. Photographic examination of the holotype of *Ch. dabryi* (MNHN 0000-5078; Fig. 5d, e) showed that the species develops a straight dorsal profile of the head, a gradually elevated humpback behind the head, fewer than 70 lateral-line scales, and a greyish-black caudal fin, all of them separating it from *Ch. oxycephalus*, as here defined (Fig. 4b).

So far synonymized with *Ch. oxycephalus* in Chinese literature is *Ch. abramoides*, a species originally described in *Culter* by Dybowski (1872) from Lake Shinkai and the Ussuri River. Our photographic examination on the holotype (ZMB 7933; Fig. 5b, c) of *Ch. abramoides* indicated that the species possesses a markedly concave dorsal profile of the head, 65 or 66 lateral-line pored scales, a protruding lower jaw greater than the upper jaw in length, dorsal spine nearly equal to HL, and longer pectoral fins with adpressed fin tip extending to or beyond pelvic-fin insertions, all of them unshared with *Ch. oxycephalus*, as here defined. Hence, we are fully in agreement with Bogutskaya and Naseka (2004) and Dyldin et al. (2023) that *Ch. abramoides* and *Ch. oxycephalus* are distinct species. A markedly concave dorsal profile of the head and a steeply uprising humpback behind the head also distinguish *Ch. abramoides* from *Ch. dabryi*. Both are further distinct in head depth and anal-fin length (Table 1). All above findings indicate that *Ch. abramoides*, *Ch. dabryi*, and *Ch. oxycephalus* are separate valid species. This study does not support that Bogutskaya et al.’s (2008) assertion that the three species might be synonyms.

### ﻿Taxonomic status of relevant species

#### ﻿*Chanodichthys dabryi shinkainensis* (Yih & Chu, 1959)

This subspecies was originally described in *Erythroculter* from Lake Shinkai of northeastern China (Yih and Chu 1959). Its establishment was based on its variation in body shape with *E. d. dabryi*. This classification has been followed widely in Chinese literature (see Zhang and Zhao 2016). Remarkable morphological differences between the two subspecies were unraveled in Zhang et al.’s (2008) morphometrics analyses. Our comparison of available specimens demonstrated that both differ in the interorbital width smaller (vs larger) than the snout length, pelvic fins reaching the anal-fin origin (vs the anus), and a silvery (vs dorsally grey-black, abdominally silvery-white) body. The syntype (IHB 58-1558; Fig. 5a) of *Ch. shinkainensis* has a steeply uprising humpback behind the head, a deep body with its maximum depth being equal to or greater than HL, pectoral fins extended backwards beyond pelvic-fin insertions, and pelvic fins reaching the anal-fin origin, these characters are diagnostic for *Ch. abramoides*. On this basis, we follow Bogutskaya et al. (2008) to consider both as the same species; *Ch. dabryi shinkainensis* is, thus, a junior synonym of *Ch. abramoides*, according to the principle of nomenclatural priority of ICZN (1999).

#### ﻿Notes on cultrin fishes from Lakes Dongting and Shinkai

Previous ichthyological identifications were based on a single specimen or even on mutilated or teratological specimens, and the original descriptions were often lacking in detail, establishing new species with minor character differences. The confusion remained unresolved owing to the unavailability of re-examination of the types (including topotypes) and the ambiguity of the original records. A thorough review of cultrin specimens by this study revealed that the so-called *Ch. oxycephalus* of the Chinese literature is actually “neither fish nor fowl” and is a combination of features displayed by several distinct species, including *Ch. oxycephaloides*, *Ch. abramoides*, and *Ch. dabryi*. This finding may also provide a rationale for the absence of any documented sampling record of *Ch. oxycephalus* from its type locality (the middle Chang-Jiang basin since 1959). In this contemporary era, the utilization of information technology facilitates more comprehensive examination of the type specimens and original descriptions, e.g. the taxonomic reviews of cultrin fishes in Lakes Dongting and Shinkai.

**Figure 6.**
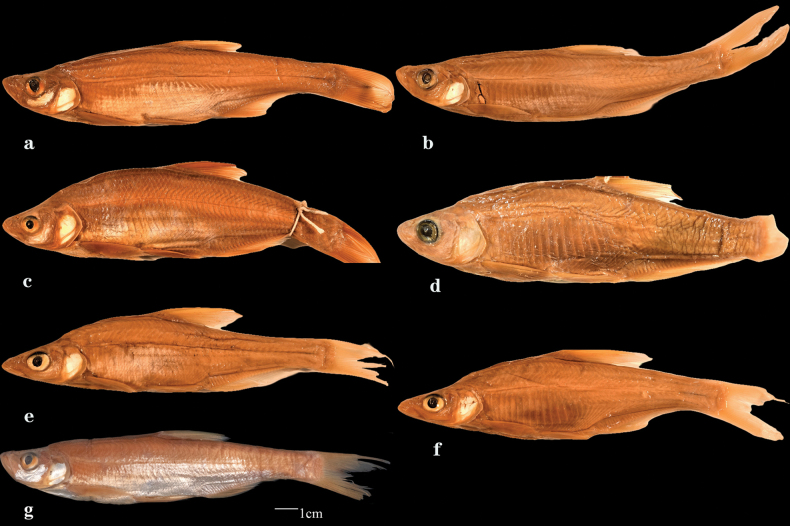
Lateral views of cultrin fish specimens collected by Clifford Pope during 1921 from Lake Dongting. a. AMNH 10842; b. AMNH 10850; c. AMNH 12166; d. AMNH 15243; e. AMNH 10871; f. AMNH 10875; g. MCZ 32640.

Lake Dongting, one of two largest river-linked subtropical lakes lying within the mid-lower Chang-Jiang basin of East China, harbors a high species diversity of cultrin fishes (Chen et al. 2022b). Nichols (1928, 1943) recorded a batch of cultrins taken by Clifford Pope in 1921 from Huping (presently Yueyang City, Hunan Province) of the lake (Fig. 6). These species were recognized as *Ch. dabryi*, *Ch. erythropterus*, *Ch. mongolicus*, *Ch. oxycephalus*, *Ch. oxycephaloides*, and *Ch. recurviceps*. Examination on specimens under the name of *Ch. recurviceps*, curated at AMNH and MCZ, found species misidentifications. *Ch. recurviceps* was initially described in *Leuciscus* by Richardson (1846) based only on a picture of a fish from Canton (today’s Guangzhou City of Guangdong Province) by Reeves (Whitehead 1970). This first-named cultrin fish is known as an economic fish in South China, including the Pearl River and Hainan Island (Pan et al. 1991; Chen et al. 1998; Yang et al. 2016; Xiang et al. 2021).

The species, represented by AMNH 10842, AMNH 10850, and MCZ 32640 (Fig. 6a, b, g) is *Ch. erythropterus*, and the specimen AMNH 12166 (Fig. 6c) belongs to *Ch. mongolicus*. The specimen AMNH 15243 (Fig. 6d), listed as *Ch. oxycephalus* is a misidentification of *Pseudobrama simoni*. Two specimens (AMNH 10871 and 10875) of *Ch. oxycephaloides* (Fig. 6e, f) represent *Ch. oxycephalus*.

Lake Shinkai, the largest freshwater body in northeast Asia, is a Sino-Russian border lake of the Amur River basin (Turanov et al. 2019). The taxonomy of cultrin fish species of this lake has long attracted attention from Chinese and Russian researchers. Currently, six species of cultrin fishes have been recorded from Lake Khanka of Russia: *Culter alburnus*, *Chanodichthys abramoides*, *Ch. dabryi*, *Ch. erythropterus*, *Ch. mongolicus*, and *Ch. oxycephalus* (Bogutskaya and Naseka 1996; Bogutskaya et al. 2008). Turanov et al.’s (2019) molecular phylogenetic analysis utilizing DNA barcoding techniques reported the occurrence of four species in this lake: *Cu. alburnus*, *Ch. erythropterus*, *Ch. mongolicus*, and *Ch. oxycephalus*, but, as we have shown above, the last species is a misidentification of *Ch. dabryi*.

Yih and Chu (1959) were the first to provide a taxonomic revision of the cultrin fish species of China, with a particular focus on Lake Shinkai (now Lake Xingkai; Appendix 1: Table A2). Six species or subspecies were recorded from this lake: *Culter compressocorpus*, *Erythroculter erythropterus*, *E. dabryi shinkainensis*, *E. ilishaeformis*, *E. mongolicus* and *E. oxycephalus*. Provided that the taxonomic confusions surrounding *E. erythropterus* and *Cu. alburnus* have been cleared up (Bogutskaya and Naseka 2004), the *E. erythropterus* of Yih and Chu is in fact a misidentification of *Cu. alburnus*. *Erythroculter ilishaeformis* is so far considered as a synonym of *Culter* (or *Erythroculter*) *alburnus* (Chen et al. 1998). Here, we follow Bogutskaya and Naseka (2004) in regarding *E. ilishaeformis* as a junior synonym of *Chanodichthys erythropterus*, a species currently misidentified as *Culter alburnus* in China (Chen et al. 1998). *Erythroculter mongolicus* is currently assigned to *Chanodichthys*, along with *E. dabryi shinkainensis* and *E. oxycephalus* (Chen et al. 1998; Zhao 2022). As noted above, *E. dabryi shinkainensis* is a junior synonym of *Ch. abramoides*. Additionally, Yih and Chu (1959) described specimens from the Sungari River of the middle Amur River (Heilong-Jiang) basin as *E. ilishaeformis sungarinensis*, which has been resolved as a junior synonym of *Cu. alburnus* (Chen et al. 1998; Zhao 2022).

## ﻿Comparative material examined

*Cu. abramoides* ZMB 7933 (1), Syntype, Ussuri River and Lake Xingkai, southeastern Russia.

*Cu. alburnus* ZIN 5585 (1), Lectotype, Rivers draining to the Gulf of Tschili, China.

*Cu. compressocorpus*IHB 58-1572~1575 (6), Dongbei-0520–0523, Syntypes, Lake Xiaoxingkai & Lake Jingpo, China.

*Cu. dabryi* MNHN 5078 (1), Holotype, Chang-Jiang, China.

*Cu. dabryi*IHB (19), Hubei, Hunan, Jiangxi, Anhui, Heilongjiang Provinces, China.

*Cu. ilishaeformis* MNHN 0000-5055 (1), Syntype, Chang-Jiang, China.

*Cu. mongolicus* ZIN 2950-51 (2), Lectotype and paralectotype: Mongolia and Manchura, northern China.

*E. dabryi shinkainensis*IHB 58-1548–1567 (21), Syntypes, Lake Daxingkai, Heilong-Jiang, China.

## ﻿Conclusions

The non-comparative description ​of *Ch. oxycephaloides*, the biased morphological description and drawing of *Ch. oxycephalus* (the count of lateral line scales) in Fauna Sinica since 1998, are considered responsible for the longstanding confusion about the relationship between *Ch. oxycephalus* and *Ch. oxycephaloides* in the studies of Zhang et al. (2008) and Wang et al. (2019). Early modern species descriptions (17^th^–18^th^ century) often contained inadequate morphological characterization, resulting in taxonomically unreliable records. These limitations were exacerbated when later scholars neglected type specimen verification, leading to entrenched misidentifications. Kreyenberg and Pappenheim (1908) failed to review types and consult the original literature, leading to the creation of “*Ch. oxycephaloides*”. Similarly, due to the scientific conditions at the time, Yih and Chu (1959) could not compare the holotypes of *Ch. oxycephalus* with those of *Ch. oxycephaloides*, and incorrectly recognized *Ch. abramoides* as a synonym of *Ch. oxycephalus* by the number of lateral-line scales. Together, these have led to more than a century of misunderstanding, which confirms the importance of type specimen and original literature for taxonomic revision (Myer 1940).

Hence, to avoid the recurrence of such confusion, ichthyologists should identify fish species based on complete specimens, with original descriptions documented in as much detail as possible, and with as much knowledge as possible of the fish’s life-history cycle (including habitat, habits, coloration, etc.). Ichthyologists need to be aware that fishes exhibit different characters at different growth periods and in different sexes, to avoid misidentifying species in terms of minor morphological differences. At the same time, the exchange of literature and specimens should be strengthened so that the same fish will not be categorized into different taxa, or different species will not be treated as the same species. The number of species with synonyms will then be reduced, as well as the misidentifications. Even if problems are stumbled upon later, the specimens can be rechecked by obtaining name-bearing types, voucher specimens, original records, accompanying drawings, and/or topotypes.

## Supplementary Material

XML Treatment for Chanodichthys oxycephalus

## ﻿Additional information

### Use of AI

No use of AI was reported.

### Author ORCIDs

Xiao Chen https://orcid.org/0000-0002-0687-7175

E Zhang https://orcid.org/0000-0002-6971-7160

### Data availability

All of the data that support the findings of this study are available in the main text.

## ﻿Appendix 1

**App References**

Basilewsky S (1855) Ichthyographia Chinae borealis. Nouveaux Mémoires de la Société Impériale des Naturalistes de Moscou 10: 215–263, pls 1–9, 467–488.

Bleeker P (1871) Memoire sur les cyprinoïdes de Chine. Verhandelingen der Koninklijke Akademie van. Amsterdam 12: 1–91, pls. 1–14.

Ding RH (1990) On a new subspecies of genus *Erythroculter* from Qionghai Lake, Sichuan, China (Cypriniformes: Cyprinidae). Acta Zootaxonomica Sinica 15(2): 246–250. [In Chinese]

Dybowski B (1872) Zur Kenntniss der Fischfauna des Amurgebietes. Verhandlungen der Kaiserlich-Königlichen Zoologisch-Botanischen Gesellschaft in Wien 22: 209–222.

He JC, Liu ZH (1980) Description of a new subspecies of *Erythroculter* from Yunnan, China. Zoological Research 1(4): 483–485. [In Chinese]

Kreyenberg M, Pappenheim P (1908) Ein Beitrag zur Kenntnis der Fische der Jangtze und seiner Zuflüsse. Sitzungsberichte der Gesellschaft Naturforschender Freunde zu Berlin 1908: 95–109. https://doi.org/10.5962/bhl.part.12852

Richardson J (1846) Report on the Ichthyology of the Seas of China and Japan. R. and J.E. Taylor, London, 153 pp. https://doi.org/10.5962/bhl.title.59530

Tirant G (1883) Mémoire sur les poissons de la rivière de Hué. 1883: 80–101. Saigon.

Yih BL, Chu ZR (1959) Review of the genera *Culter* and *Erythroculter* of China. Acta Hydrobiologica Sinica 3(2): 170–196. [In Chinese]

**Table A1.** List of valid species of genus *Chanodichthys* and *Culter* and their related information.

Valid name	Name used	Original reference	Type locality	Geographical area
*Chanodichthys abramoides*	*Culter abramoides*	Dybowski 1872	Lake Shinkai and the Ussuri River	East Asia
*Chanodichthys dabryi*	*Culter dabryi*	Bleeker 1871	Chang-Jiang, China	East Asia
*Chanodichthys erythropterus*	*Culter erythropterus*	Basilewsky 1855	Rivers flowing into Bohai Bay, Beijing, China	East Asia
*Chanodichthys flavipinnis*	*Culter flavipinnis*	Tirant 1883	Hué, Vietnam	South Asia: Vietnam
*Chanodichthys mongolicus*	*Leptocephalus mongolicus*	Basilewsky 1855	Mongolia and Manchuria, northern China	East Asia: Vietnam, China, Russia, Mongolia, Korea and Japan
*Chanodichthys elongatus*	*Erythroculter mongolicus elongatus*	He and Liu 1980	Lake Chenghai, Yunnan Province, China	Lake Chenghai
*Chanodichthys qionghaiensis*	*Erythroculter mongolicus qionghaiensis*	Ding 1990	Lake Qionghai, Sichuan Province, China	East Asia
*Chanodichthys oxycephaloides*	*Culter oxycephaloides*	Kreyenberg and Pappenheim 1908	Lake Dongting, Hunan Province, China	Chang-Jiang
*Chanodichthys oxycephalus*	*Culter oxycephalus*	Bleeker 1871	Chang-Jiang, China	Chang-Jiang
*Chanodichthys recurviceps*	*Leuciscus recurviceps*	Richardson 1846	Guangzhou, China	East Asia: China
*Culter alburnus*	*Erythroculter ilishaeformis*	Basilewsky 1855	Rivers flowing into Bohai Bay, Beijing, China	East Asia: Mongolia, Russia, southern and eastern China, and Vietnam
*Culter compressocorpus*	*Cultrichthys compressocorpus*	Yih and Chu 1959	Lake Xingkai, China	Lake Xingkai, China

**Table A2.** Details on the fish collections mentioned in this paper.

Abbreviation / name	Preserved unit / other names
**AMNH**	American Museum of Natural History, New York, USA
**IHB**	Institute of Hydrobiology, Chinese Academy of Sciences, Wuhan, China
**MCZ**	Museum of Comparative Zoology, Harvard University, Cambridge, Massachusetts, USA
**MNHN**	Muséum National d’Histoire naturelle, Systématique et Évolution, Laboratoire d’Ichthyologie Générale et Appliquée, Paris, France. Other Abbr.: MNHN-IC
**ZIN**	Laboratory of Ichthyology, Zoological Institute, Russian Academy of Sciences, St. Petersburg, Russia. Other Abbr.: ZIAS, ZISP, ZIL. Old Name: Academy of Sciences of the USSR
**ZMB**	Museum für Naturkunde, Leibniz-Institut für Evolutions- und Biodiversitätsforschung, Berlin, Germany. Other Abbr.: ISZZ, ZMHU. Old Name: Humboldt-Universität, Museum für Naturkunde, Zoologisches Museum, Vertebraten (Wirbeltiere), Ichthyologie, Berlin, Germany
**ASIZB**	Institute of Zoology, Chinese Academy of Sciences, Beijing, China Other Abbr.: ZMFMIB. Old Name: Zoologial Museum, Fan Memorial Institute of Biology, Tsinghua University, Beijing, China
**Chang-Jiang**	Yangtze River or Yang-tse-kiang
**Heilong-Jiang**	Heilongjiang River or Amur River
**Lake Dongting**	Tungtingsee or Lake Tungting
**Lake Xingkai**	Lake Khanka, Lake Chanka or Lake Shinkai, including Lake Xiaoxingkai (Lake Little Khanka) and Lake Daxingkai (Lake Big Khanka).
